# Supplementary material for: Quantum State Optimization and Computational Pathway Evaluation for Gate-Model Quantum Computers
Source: Sci Rep. 2020 Mar 11;10:4543. doi: 10.1038/s41598-020-61316-4 (PMC7066182; doi:10.1038/s41598-020-61316-4)
Supplement: Supplementary file 1 — Supplemental information. [file 41598_2020_61316_MOESM1_ESM.pdf]

Quantum State Optimization and Computational Pathway Evaluation for Gate-Model Quantum Computers

Laszlo Gyongyosi[1,2,3,*]

[1]School of Electronics and Computer Science, University of Southampton, Southampton, SO17 1BJ, UK
[2]Department of Networked Systems and Services, Budapest University of Technology and Economics, Budapest, H-1117 Hungary
[3]MTA-BME Information Systems Research Group, Hungarian Academy of Sciences, Budapest, H-1051 Hungary
[*]gyongyosi@hit.bme.hu

ABSTRACT

A computational problem fed into a gate-model quantum computer identifies an objective function with a particular computational pathway (objective function connectivity). The solution of the computational problem involves identifying a target objective function value that is the subject to be reached. A bottleneck in a gate-model quantum computer is the requirement of several rounds of quantum state preparations, high-cost run sequences, and multiple rounds of measurements to determine a target (optimal) state of the quantum computer that achieves the target objective function value. Here, we define a method for optimal quantum state determination and computational path evaluation for gate-model quantum computers. We prove a state determination method that finds a target system state for a quantum computer at a given target objective function value. The computational pathway evaluation procedure sets the connectivity of the objective function in the target system state on a fixed hardware architecture of the quantum computer. The proposed solution evolves the target system state without requiring the preparation of intermediate states between the initial and target states of the quantum computer. Our method avoids high-cost system state preparations and expensive running procedures and measurement apparatuses in gate-model quantum computers. The results are convenient for gate-model quantum computations and the near-term quantum devices of the quantum Internet.

A Appendix

A.1 Abbreviations

NISQ Noisy Intermediate-Scale Quantum

QAOA Quantum Approximate Optimization Algorithm

RKHS Reproducing Kernel Hilbert Space

A.2 Notations

The notations of the manuscript are summarized in Table A.1.

Table A.1. Summary of notations.

Notation	*Description*
QG	Quantum gate structure of a gate-model quantum computer.
$U_i(\theta_i)$	An i-th unitary gate, $U_i(\theta_i) = \exp(-i\theta_i P_i)$, where P_i is a generalized Pauli operator formulated by a tensor product of Pauli operators $\{X,Y,Z\}$, while θ_i is referred to as the gate parameter associated to $U_i(\theta_i)$.
$\|\vec{\theta}\rangle$	System state of the quantum computer, $\|\vec{\theta}\rangle = U_L(\theta_L)U_{L-1}(\theta_{L-1})\ldots U_1(\theta_1)$, where $U_i(\theta_i)$ identifies an i-th unitary gate.
$\vec{\theta}$	Gate parameter vector, a collection of gate parameters of the L unitaries, $\vec{\theta} = \theta_L, \theta_{L-1}, \ldots, \theta_1$.
$C(z)$	Objective function of a computational problem fed into the quantum computer. It identifies the computational pathway (connectivity of the objective function as) $C(z) = \sum_{ij\in QG} C_{ij}(z)$, where $C_{ij}(z)$ is evaluated between quantum states ij in the QG structure of the gate-model quantum computer.
$C^*(z)$	Computational pathway in the target state $\|\vec{\theta}^*\rangle$.
z	A bitstring.
$f(\vec{\theta})$	Objective function.
$f^*(\vec{\theta})$	A target objective function value.
$\|\vec{\theta}_0\rangle$	Initial system state of the quantum computer.
$\|\vec{\theta}^*\rangle$	Target system state of the quantum computer subject to be determined that achieves $f^*(\vec{\theta})$.
χ	Vector of regression coefficients.
$\vec{\theta}_0$	Collection of gate parameters in the $\|\vec{\theta}_0\rangle$ initial system state.
$\vec{\theta}^*$	Collection of gate parameters in the $\|\vec{\theta}^*\rangle$ target system state.
$F(\vec{\theta}_0)$	Component of $\vec{\theta}_0$.
$F(U)$	Fixed component for an arbitrary $\vec{\theta}$.
$+$	Moore–Penrose pseudoinverse.
$\mathscr{G}$	Connectivity graph, $\mathscr{G} = (V,S)$, with a set V of vertexes, and a set S of arcs.
V	Set of vertexes in $\mathscr{G}$.
S	Set of arcs in $\mathscr{G}$.
v	A vertex of V the $\mathscr{G}$ environmental graph.

$s_{i,j}$	Edge $s_{i,j}$ with index pair (i,j), it identifies a connection between nodes v_i and v_j.
$\mathscr{X}$	Input space.
$\mathscr{K}$	Kernel machine.
$\mathscr{H}$	Reproducing Kernel Hilbert Space (RKHS) associated with the kernel machine $\mathscr{K}$.
Γ	A nonlinear map, $\Gamma : \mathscr{X} \to \mathscr{H}$, from $\mathscr{X}$ to the high-dimensional Hilbert space $\mathscr{H}$ associated with $\mathscr{K}$.
$\vec{\kappa}$	Vector of initial $s_{i,j}$ edges in the $\mathscr{G}$ connectivity graph.
$\vec{\Omega}$	Vector of the actual $C_{s_{i,j}}(z)$ objective function values, associated to the $s_{i,j}$ edges in the $\mathscr{G}$ connectivity graph.
κ_i	An i-th element of $\vec{\kappa}$.
Ω_{κ_i}	An i-th element of $\vec{\Omega}$.
Υ_0	Initial element in the input space $\mathscr{X}$, defined as $\Upsilon_0 = (\vec{\kappa}, \vec{\Omega})^T$.
τ_0	Map of Υ_0 in $\mathscr{H}$, $\tau_0 = \Gamma(\Upsilon_0) = \lambda \vec{\theta}_0$, where λ is a matrix of eigenvectors associated with the edge and objective function values in $\lvert\vec{\theta}_0\rangle$.
Υ^*	Target element in $\mathscr{X}$ subject to be determined, $\Upsilon^* = (\vec{\kappa}^*, \vec{\Omega}^*)^T$.
$\vec{\kappa}^*$	Target vector, identifies the connectivity of the $C^*_{s_{i,j}}(z)$ objective function values in the target state $\lvert\vec{\theta}^*\rangle$.
$\vec{\Omega}^*$	Target vector, identifies the connectivity of the $C^*_{s_{i,j}}(z)$ objective function values in the target state $\lvert\vec{\theta}^*\rangle$.
κ_i^*	An i-th element of $\vec{\kappa}^*$ and $\vec{\Omega}^*$.
$\Omega^*_{\kappa_i^*}$	An i-th element of $\vec{\Omega}^*$.
τ^*	Map of the target $\Upsilon^* \in \mathscr{X}$, $\tau^* = \Gamma(\Upsilon^*) = \lambda^* \vec{\theta}^*$, where λ^* is a matrix of eigenvectors associated with the edge and objective function values in state $\lvert\vec{\theta}^*\rangle$.
$\mathscr{P}$	Projector in $\mathscr{H}$.
Υ_0	Initial element in $\mathscr{X}$.
Υ_i	Training data in $\mathscr{X}$.
Υ^*	Target element in $\mathscr{X}$.
V	An eigenvector.
β_i	Projections in $\mathscr{H}$, $\beta_i = \sum_{j=1}^{N} \alpha_j^i \mathscr{K}(\Upsilon^*, \Upsilon_j)$, where α_i is an i-th coefficient in the eigenvector V, $V = \sum_{i=1}^{N} \alpha_i \tau_i$, where τ_i is the map of training data Υ_i, $\tau_i = \Gamma(\Upsilon_i)$.
$f_d(x,y)$	Distance function in $\mathscr{H}$, $f_d(x,y) = \lVert x-y \rVert^2$.
Φ	A non-negative regularization parameter.
ζ	Terms independent of Υ^*.
ℓ_i	Parameter, $\ell_i = \sum_{k=1}^{n} \beta_k \alpha_i^k$, where n is associated to the projection $\mathscr{P}(\tau_0)$.
$\varepsilon(\Upsilon^*)$	Extremum of Υ^*, $\varepsilon(\Upsilon^*) = \frac{1}{\sum_j \sigma_j} \sum_i \Upsilon_i \sigma_i$, where $\sigma_i = \ell_i \mathscr{K}'(\varepsilon(\Upsilon^*), \Upsilon_i)$.
$\nabla_{\varepsilon(\Upsilon^*)}(f_d(\cdot))$	Gradient with respect to $\varepsilon(\Upsilon^*)$.
$f_d^{(i)}(\cdot)$	Distance function associated to an i-th iteration step.

$\mathscr{S}_{\mathscr{X}}$	Training set of N training data in $\mathscr{X}$, $\mathscr{S}_{\mathscr{X}} = \{\Upsilon_1, \ldots, \Upsilon_N\}$.
$\mathscr{S}_{\mathscr{H}}$	Set of maps of the training data in $\mathscr{H}$, $\mathscr{S}_{\mathscr{H}} = \{\Gamma(\Upsilon_1), \ldots, \Gamma(\Upsilon_N)\} = \{\tau_1, \ldots, \tau_N\}$.
R	Iteration number.
Υ_r^*	Target value Υ^* associated with an r-th iteration step, $r = 1, \ldots, R$.
Υ_R^*	Solution determined in the R-th iteration step, $\tau_R^* = \Gamma(\Upsilon_R^*)$.
